# Characteristics of gut microbiota in pregnant hepatitis b virus carriers and their correlation with liver biochemical indicators

**DOI:** 10.3389/fcimb.2026.1782685

**Published:** 2026-05-12

**Authors:** Tao Xie, Hui Wang, Jiawen Yang, Congcong Sun, Dan Li

**Affiliations:** 1 Department of Ultrasound, University-Town Hospital of Chongqing Medical University, Chongqing, China; 2 Department of Pathology, Faculty of Basic Medicine, Chongqing Medical University, Chongqing, China; 3 Medical Sciences Research Center, University-Town Hospital of Chongqing Medical University, Chongqing, China; 4 Molecular Medicine Diagnostic and Testing Center, Chongqing Medical University, Chongqing, China; 5 Laboratory of Neuropsycholinguistics, Chongqing Medical University, Chongqing, China; 6 Department of Gynaecology and Obstetrics, University-Town Hospital of Chongqing Medical University, Chongqing, China; 7 Department of Pathology, The First Affiliated Hospital of Chongqing Medical University, Chongqing, China

**Keywords:** biomedical indicators, gut microbiota, hepatitis B virus, liver function, pregnancy

## Abstract

**Background and aims:**

The stability of liver function in pregnant hepatitis B virus (HBV) carriers is closely related to both maternal and infant outcomes. However, the role of gut microbiota in HBV infection during pregnancy remains unclear. This study aimed to analyze the compositional characteristics of gut microbiota in pregnant HBV carriers and to explore their associations with liver biochemical indicators.

**Methods:**

This study enrolled 20 pregnant women with hepatitis B virus infection (HBV group) and 13 healthy pregnant women (control group, CON). Clinical data were collected from all subjects. Fresh fecal samples were obtained and subjected to 16S rRNA gene high-throughput sequencing to characterize gut microbial alpha diversity, beta diversity, and between-group differences in community composition. Spearman correlation analysis was employed to assess associations between gut microbiota and liver biochemical indicators as well as HBV DNA load.

**Results:**

Beta diversity analysis indicated a significant separation of overall microbiota structure between the HBV and CON groups. At the phylum level, the gut microbiota in both groups was predominantly composed of Bacteroidota, Firmicutes, and Proteobacteria. At the genus level, the dominant genera were *Bacteroides*, *Prevotella*, and *Faecalibacterium*. Compared with the CON group, the HBV group showed significantly decreased abundances of Synergistota and Desulfobacterota at the phylum level, Synergistia and Desulfovibrionia at the class level, and *NK4A214_group* and *Desulfovibrio* at the genus level (all *p* < 0.05), whereas *Acidaminococcus* was significantly enriched (*p* < 0.05). Spearman analysis showed that *Bacteroides, Bifidobacterium, Lachnospira, Muribaculum, Parabacteroides, Parasutterella, [Eubacterium] eligens group*, and *Prevotella* were negatively correlated with liver function-related indicators (*p* < 0.05). In contrast, *Coprococcus*, *Prevotella*, and *Ruminococcus* were positively correlated with liver function-related indicators (*p* < 0.05). Notably, *Escherichia-Shigella* demonstrated positive correlations with both liver function indicators and HBV-DNA levels (*p* < 0.05).

**Conclusions:**

Pregnant HBV carriers exhibit distinct gut microbiota compositional features compared to healthy pregnant controls. Specific bacterial genera show correlations with HBV DNA levels and liver biochemical parameters.

## 1 Introduction

Hepatitis B virus (HBV) infection remains a major global public health challenge. According to World Health Organization (WHO) statistics, approximately 296 million individuals worldwide are chronically infected with HBV, and over 820,000 deaths occur annually due to complications such as HBV-related cirrhosis and hepatocellular carcinoma (World Health Organization, 2024). Pregnant HBV carriers represents a unique population: their viral replication status and liver function stability not only directly affect maternal pregnancy outcomes but can also increase neonatal infection through mother-to-child transmission, posing a serious threat to child health (Yin et al., 2024; Yan et al., 2025). Therefore, in-depth exploration of the regulatory mechanisms of liver function in pregnant HBV carriers and the search for potential intervention targets are of clinical significance for improving maternal and infant prognosis.

Imbalance in gut microbial community structure and metabolic function homeostasis has been confirmed to be closely associated with the onset and progression of multiple liver diseases, including non-alcoholic fatty liver disease, cirrhosis, and viral hepatitis (Albillos et al., 2020). It has been reported that individuals with chronic HBV exhibit significant gut microbiota dysbiosis, characterized by a reduction in beneficial commensals alongside an expansion of potentially pathogenic taxa, and the severity of this dysbiosis has been reported to correlate positively with hepatic inflammatory activity and fibrosis stage (Wang F. et al., 2025). Other studies have shown that the gut microbiota has been reported to be associated with hepatic immune responses and hepatocyte injury–repair processes via microbe-derived metabolites. When microbial imbalance occurs, intestinal barrier function is impaired, allowing endotoxin to translocate into the circulation and activate Toll-like receptor signaling pathways, thereby exacerbating liver inflammation and contributing to HBV replication and the process of liver fibrosis (An et al., 2022).

Pregnancy entails dramatic hormonal changes, reduced gastrointestinal motility, and an immunosuppressed state, which collectively predispose the gut microbiota to structural remodeling. These shifts frequently present as reduced microbial diversity and alterations in the Firmicutes/Bacteroidetes ratio (Yuan et al., 2025). This pregnancy-specific microbiota dysbiosis is associated not only with pregnancy complications such as gestational diabetes mellitus and pre-eclampsia, but may also perturb liver function and metabolism (Zuo et al., 2025). However, research on the characteristics of gut microbiota in pregnant HBV carriers remains limited. Existing studies have primarily focused on the association between HBV infection and gut microbiota in non-pregnant populations. In pregnancy, how the HBV-carrying state affects gut microbiota structure, and whether microbiota dysbiosis correlates with liver biochemical indicators, remain to be clarified.

To address this gap, the present study recruited pregnant HBV carriers and healthy pregnant controls, using 16S rRNA high-throughput sequencing to analyze differences in alpha diversity, beta diversity, and community composition of gut microbiota between the two groups. Integrating sequencing data with clinical laboratory measurements, we further investigated the correlation between gut microbiota characteristics and liver biochemical indices as well as HBV DNA titers in pregnant HBV carriers. The goal is to clarify the contribution of gut microbiota dysbiosis to HBV-related hepatic dysfunction during pregnancy, thereby providing new insights into the clinical management and prognosis improvement of HBV infection in pregnancy.

## 2 Methods

### 2.1 Study subjects

Pregnant women who were HBV carriers (HBV group) and healthy pregnant women (control group, CON) undergoing routine prenatal examinations at the University-Town Hospital of Chongqing Medical University between January 2024 and December 2024 were enrolled as study subjects. This study represents a pilot investigation with a limited sample size, aiming to generate preliminary data and hypotheses for future larger-scale studies. The study protocol complied with the ethical standards of the Declaration of Helsinki and was approved by the institutional Ethics Committee of the hospital.

#### 2.1.1 Inclusion criteria

① Age 18–45 years. ② Diagnosis of HBV carrier conformed to the criteria specified in the Guidelines for the Prevention and Treatment of Chronic Hepatitis B (2022 version) (Hepatology C S O and Diseases C S O I, 2022). Herein, HBV carrier is defined as an individual who is seropositive for hepatitis B surface antigen (HBsAg) but remains asymptomatic with no clinical manifestations of liver disease, particularly persistently normal Alanine Aminotransferase (ALT) levels. ③ No history of using medications affecting gut microbiota (e.g., antibiotics, probiotics, prebiotics, or other medications affecting gut microbiota) within three month prior to enrollment. ④ No history of other liver diseases or conditions known to affect gut microbiota.

#### 2.1.2 Exclusion criteria

① A history of chronic metabolic diseases or active infectious diseases. ② Presence of other major systemic diseases or psychiatric disorders. ③ Presence of pregnancy-related complications, such as hypertension, diabetes mellitus, thyroid dysfunction, etc. ④ Withdrawal during the study period or refusal to continue participation for any reason. ⑤Current smoking or alcohol consumption during pregnancy, or a recent history of infection (within one month prior to enrollment).

### 2.2 Collection of demographic information and laboratory indicators

Maternal age, education level, gravidity, parity, mode of delivery, gestational weight gain, and blood pressure were collected. A 5 mL sample of morning fasting venous blood was collected from each subject. After coagulation, the sample was centrifuged at 3000 rpm for 10 min, and the separated serum was stored at -20 °C. Levels of ALT, Aspartate Aminotransferase (AST), Gamma-Glutamyl Transferase (GGT), Alkaline Phosphatase (ALP), Total Bile Acid (TBA), Total Bilirubin (TBIL), Glucose (GLU), Serum Calcium (Ca), Direct Bilirubin (DBIL), Indirect Bilirubin (IBIL), Total Protein (TP), Albumin (ALB), and Globulin (GLB) were measured using a fully automated biochemical analyzer (Model LX-20; Beckman, Fullerton, USA). For all HBV-infected participants, HBsAg, hepatitis B surface antibody (HBsAb), hepatitis B e antigen (HBeAg), hepatitis B e antibody (HBeAb), hepatitis B core antibody (HBcAb), and serum HBV DNA load were measured.

### 2.3 Stool sample collection

On the day prior to stool collection, subjects were instructed to abstain from alcohol and medications and to fast for 8 hours. Fresh stool samples were collected using disposable sterile samplers (Registration No. 20200208, Made in China) containing a proprietary preservation solution. According to the manufacturer’s instructions, this preservation solution is formulated to completely inhibit microbial activity through a proprietary fixative and is suitable for room-temperature preservation of microbial or tissue samples intended for DNA or RNA analysis. Stool sample collection and preservation were strictly performed in accordance with the manufacturer’s instructions. Samples were promptly placed into the samplers, sealed, and rapidly transferred to a –80 °C freezer for storage until further use.

### 2.4 Genomic DNA extraction and amplification

Total genomic DNA was extracted from the samples using the CTAB/SDS method. DNA concentration and purity were analyzed by agarose gel electrophoresis, and the DNA concentration was standardized to 1 ng/μL with sterile water. The V3-V4 region of the 16S rRNA gene was amplified using the specific primers 338F (5’-ACTCCTACGGGAGGCAGCAG-3’) and 806R (5’-GGACTACHVGGGTWTCTAAT-3’). All PCR reactions were performed using Phusion^®^ High-Fidelity PCR Master Mix. The final library was sequenced on the Illumina NovaSeq 6000 platform, generating 250 bp paired-end reads.

### 2.5 High-throughput sequencing analysis

Raw sequencing data were subjected to quality filtering using Trimmomatic v0.36. A sliding window approach was applied with a window size of 50bp and an average quality threshold of 20. Reads shorter than 120bp after trimming were discarded. Clean reads were then merged using Pear v0.9.6 with a minimum overlap of 10 bp and a mismatch ratio of 0.1. Chimeric sequences were detected and removed using the UCHIME algorithm implemented in Vsearch v2.7.1. After quality control, fastQC v0.11.9 was used to evaluate sequencing quality. The read length distribution after trimming was assessed to confirm the consistency of the amplified target region. The gene sequences were assigned against the SILVA database using the RDP classifier algorithm to determine the taxonomic classification of each sequence in the samples. The relative abundance of each bacterial taxon in the samples was recorded. Operational Taxonomic Units (OTUs) were clustered using Mothur software with a 97% similarity threshold. Bar graphs depicting the composition of fecal microbiota were generated, and the dominant flora were analyzed. Alpha diversity indices were calculated based on OTUs information using QIIME 1.9.0 software. Beta diversity was assessed and compared using Principal Co-ordinates Analysis (PCoA), Principal Component Analysis (PCA), Partial Least Squares Discriminant Analysis (PLS-DA), and Non-metric Multidimensional Scaling (NMDS), based on the relative abundance of microbiota in the samples. Metagenomic functional profiles were inferred from the 16S rRNA sequences using PICRUSt. The metabolic functions of the microbiota, such as amino acid, carbohydrate, lipid, cofactor, and vitamin synthesis, were predicted by aligning the gene sequencing results with the Kyoto Encyclopedia of Genes and Genomes (KEGG) database.

### 2.6 Statistical analysis

Statistical analyzes were performed using SPSS 23.0 software and R version 4.0.5. For continuous variables, normality was assessed using the Shapiro–Wilk test. Normally distributed variables are presented as mean ± standard deviation(`X ± s) and were compared between groups using the independent-samples t-test. Non-normally distributed continuous variables are reported as median (Q1, Q3) and were analyzed using appropriate non-parametric tests. Categorical variables are presented as number (percentage) and were compared using the chi-square test or Fisher’s exact test.

For gut microbiota analysis, alpha diversity indices (ACE, Chao1, Shannon, InvSimpson, PD_whole_tree, Goods_coverage, Richness, Simpson, and Pielou) were compared between the two groups using the Wilcoxon rank-sum test. Beta diversity was assessed based on Bray-Curtis distance matrices, and differences in microbial community structure between groups were tested using Permutational Multivariate Analysis of Variance (PERMANOVA) with 999 permutations.

Differential abundance analysis of microbial taxa at the phylum, class, order, family, and genus levels was performed using the Wilcoxon rank-sum test. To correct for multiple comparisons, the Benjamini-Hochberg false discovery rate (FDR) method was applied, and taxa with an FDR-adjusted *p*-value < 0.05 were considered significantly different between groups.

Spearman’s rank correlation analysis was used to assess associations between the relative abundance of gut microbiota at the genus level and liver biochemical indicators, as well as HBV DNA load. Correlation coefficients (r) and FDR-adjusted p-values were calculated, and correlations with *p* < 0.05 were considered statistically significant.

## 3 Results

### 3.1 Comparison of general characteristics between the two groups

According to the inclusion and exclusion criteria, 20 pregnant HBV carriers and 13 healthy pregnant women were finally included. Regarding HBV infection-related indicators in the HBV group, the median serum HBV DNA load was 2159.97 (190, 1256) IU/mL. All 20 HBV-infected participants (100%) tested positive for HBsAg and HBcAb. HBeAg was positive in 4 participants (20%), while HBeAb was positive in 17 participants (85%). Only one participant (5%) tested positive for HBsAb. The general characteristics of the two groups are shown in **Table 1**. There were no statistically significant differences between the two groups in terms of age, education level, gravidity, parity, mode of delivery, maternal and neonatal weight, systolic blood pressure, diastolic blood pressure, blood glucose, serum calcium, or liver function-related indicators.

**Table 1:** Comparison of general characteristics between the two groups.

Variable	CON group (n=13)	HBV group (n=20)	Statistical value	*P*-value
Age [`X ± s, years]	29.0 ± 2.9	29.7 ± 3.3	-0.572	0.572
Education Level [n (%)]			1.060	0.588
High School/Technical Secondary School	2 (15.38)	2 (10)		
Bachelor’s Degree/College	10 (76.92)	17 (85)		
Master’s Degree	1 (7.69)	1 (5)		
Gravidity [M (Q1, Q3), times]	2.0 (1.0, 2.0)	2.0 (1.0, 3.0)	-0.640	0.522
Parity [M (Q1, Q3), times]	0.0 (0.0, 1.0)	0.0 (0.0, 1.0)	-1.404	0.160
Pre-delivery Weight [`X ± s,kg]	53.3 ± 8.4	52.9 ± 7.9	0.164	0.871
Mode of Delivery [n (%)]			1.193	0.275
Vaginal Delivery	9 (69.23)	10 (50)		
Cesarean Section	4 (30.77)	10 (50)		
Weight Gain [`X ± s,kg]	15.8 ± 2.9	13.6 ± 4.6	1.559	0.129
Neonatal Weight [M (Q1, Q3), kg]	3320(3110, 3443)	3225 (3074, 3786)	-0.331	0.741
HBV DNA [M (Q1, Q3), IU/mL]	–	2159.97 (190,1256)	–	–
HBs-Ag Positive [n (%)]	–	20 (100)	–	–
HBs-Ab Positive [n (%)]	–	1 (5)	–	–
HBe-Ag Positive [n (%)]	–	4 (20)	–	–
HBe-Ab Positive [n (%)]	–	17 (85)	–	–
HBc-Ab Positive [n (%)]	–	20 (100)	–	–
SBP [`X ± s, mm Hg]	119.0 ± 12.6	116.8 ± 10.1	0.556	0.582
DBP [`X ± s, mm Hg]	74.9 ± 9.4	73.8 ± 8.4	0.363	0.719
GLU [`X ± s, mmol/L]	4.74 (4.53, 5.13)	4.91 (4.38, 5.57)	-0.370	0.711
Ca [`X ± s, mmol/L]	2.26 ± 0.11	2.31 ± 0.14	-1.036	0.308
ALT [M (Q1, Q3),U/L]	12.1 (8.5, 13.7)	13.8 (10.2, 18.5)	-0.837	0.403
AST [M (Q1, Q3),U/L]	16.1 (14.3, 18.6)	18.3 (17.5, 23.3)	-1.849	0.064
ALP [`X ± s,U/L]	179.5 ± 49.3	163.9 ± 56.4	0.794	0.434
GGT [M (Q1, Q3),U/L]	13.3 (10.3, 16.1)	14.6 (9.9, 20.7)	-0.195	0.846
TBA [M (Q1, Q3),μmol/L]	4.0 (3.1, 5.0)	3.2 (1.8, 5.6)	-0.370	0.711
TBIL [M (Q1, Q3),μmol/L]	9.30 (7.76, 10.12)	9.32 (7.20, 11.21)	-0.211	0.833
DBIL [M (Q1, Q3),μmol/L]	1.71 ± 0.62	2.03 ± 0.91	-1.064	0.296
IBIL [M (Q1, Q3),μmol/L]	7.42 (6.11, 8.43)	6.99 (5.51, 8.59)	-0.234	0.815
TP [M (Q1, Q3),g/L]	60.50 (58.78, 62.56)	62.07 (60.55, 66.66)	-1.616	0.106
ALB [`X ± s,g/L]	35.9 ± 2.2	36.9 ± 3.6	-0.845	0.405
GLB [`X ± s,g/L]	25.30 ± 2.09	26.07 ± 3.25	-0.738	0.466

SBP, Systolic Blood Pressure; DBP, Diastolic Blood Pressure.

### 3.2 Quality control results of 16S rRNA gene sequencing of gut microbiota

**Figure 1:**
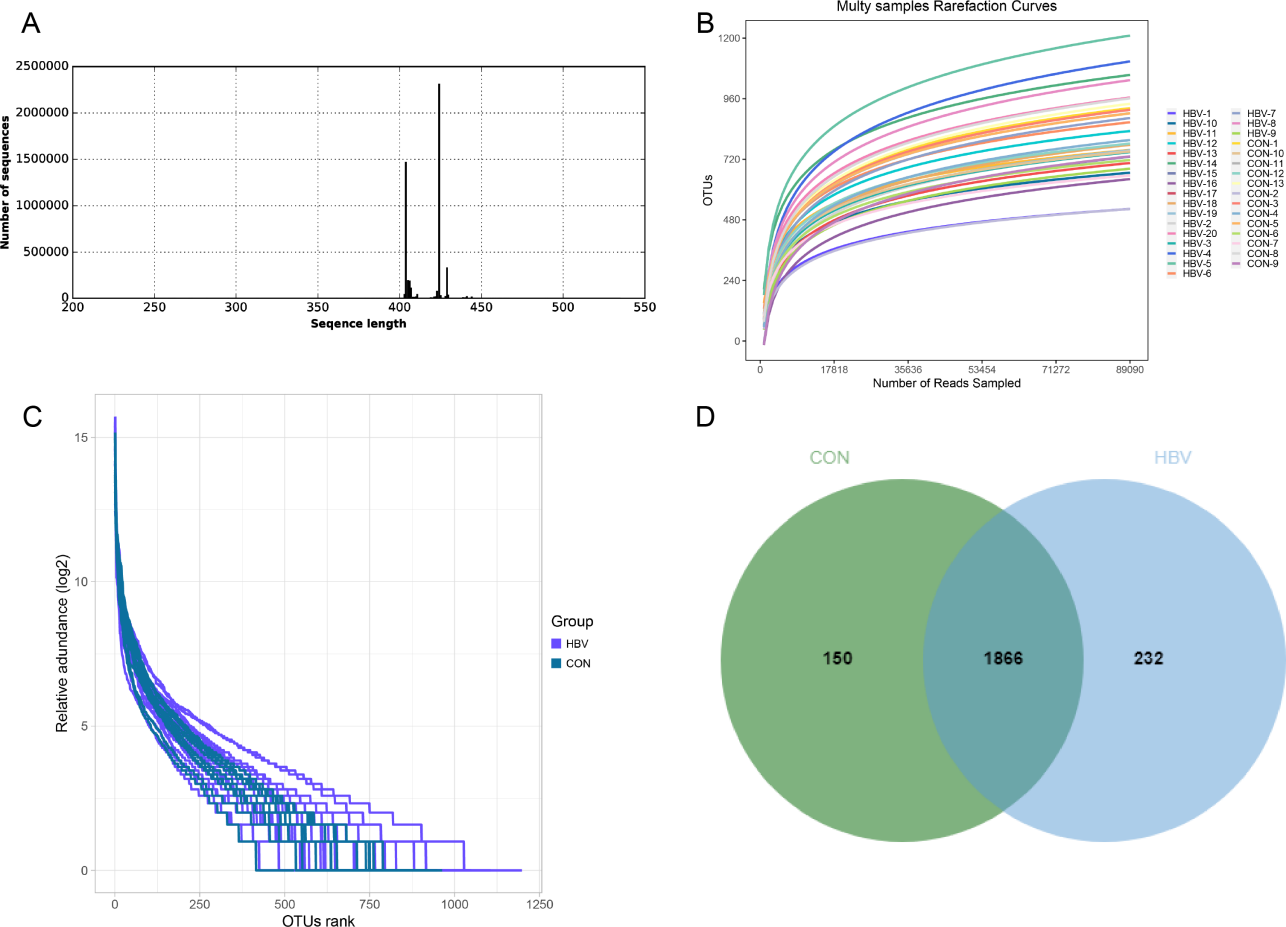
Quality control results of 16S rRNA gene sequencing of gut microbiota. **(A)** Distribution of high-quality sequences; **(B)** Rarefaction curve; **(C)** Rank abundance curve; **(D)** Venn diagram of OTU distribution.

After processing the Fastq data, valid sequences were obtained, with length distributions ranging from 400 to 450 bp, consistent with the theoretical length of the 16S V3–V4 region, indicating that the detected sequences met the requirements (**Figure 1A**). Rarefaction curve analysis showed that as the sample size increased, the curves tended to plateau, indicating that the sequencing data volume was saturated and could cover the vast majority of microbial information in the samples (**Figure 1B**). The rank abundance curves gradually became flat, suggesting high species richness and relatively even distribution (**Figure 1C**). These results indicate that the sequencing results were valid and reliable, allowing for subsequent analyzes. A Venn diagram showed the common and unique ASVs between the two groups of pregnant women. There were 2248 ASVs shared between the two groups, with 150 unique ASVs in the CON group and 232 unique ASVs in the HBV group, and 1866 ASVs were shared between the two groups (**Figure 1D**).

### 3.3 Alpha diversity analysis of gut microbiota in the two groups

**Figure 2:**
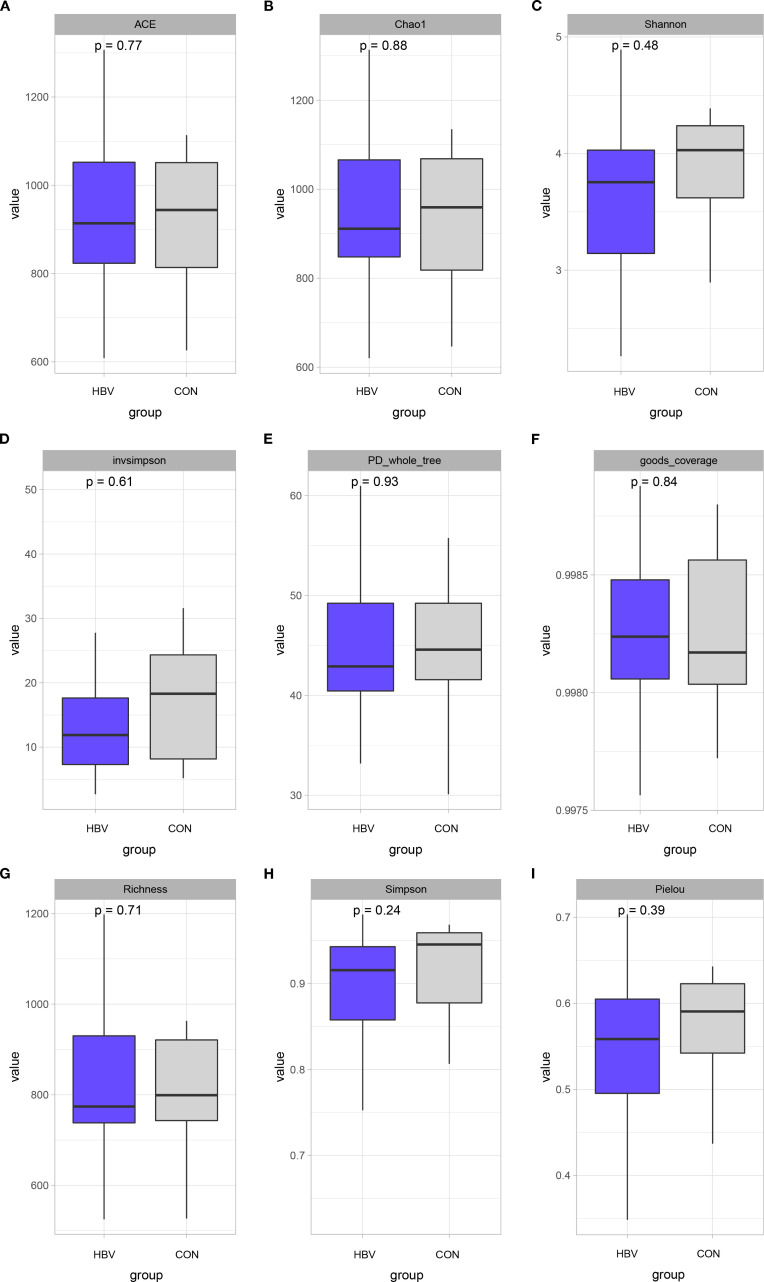
Alpha diversity analysis of gut microbiota in the two groups. **(A)** ACE analysis; **(B)** Chao1 analysis; **(C)** Shannon analysis; **(D)** InvSimpson analysis; **(E)** PD_whole_tree analysis; **(F)** Goods_coverage analysis; **(G)** Richness analysis; **(H)** Simpson analysis; **(I)** Pielou analysis.

The Wilcoxon rank-sum test was used to compare alpha diversity indices between the two groups. The results showed no statistically significant differences between the HBV and CON groups in ACE (*p* = 0.77), Chao1 (*p* = 0.88), Shannon (*p* = 0.48), InvSimpson (*p* = 0.61), PD_whole_tree (*p* = 0.93), Goods_coverage (*p* = 0.84), Richness (*p* = 0.71), Simpson (*p* = 0.24), or Pielou (*p* = 0.39) indices (**Figures 2A–I**), indicating comparable within-sample gut microbiota diversity between the two groups.

### 3.4 Beta diversity analysis of gut microbiota in the two groups

**Figure 3:**
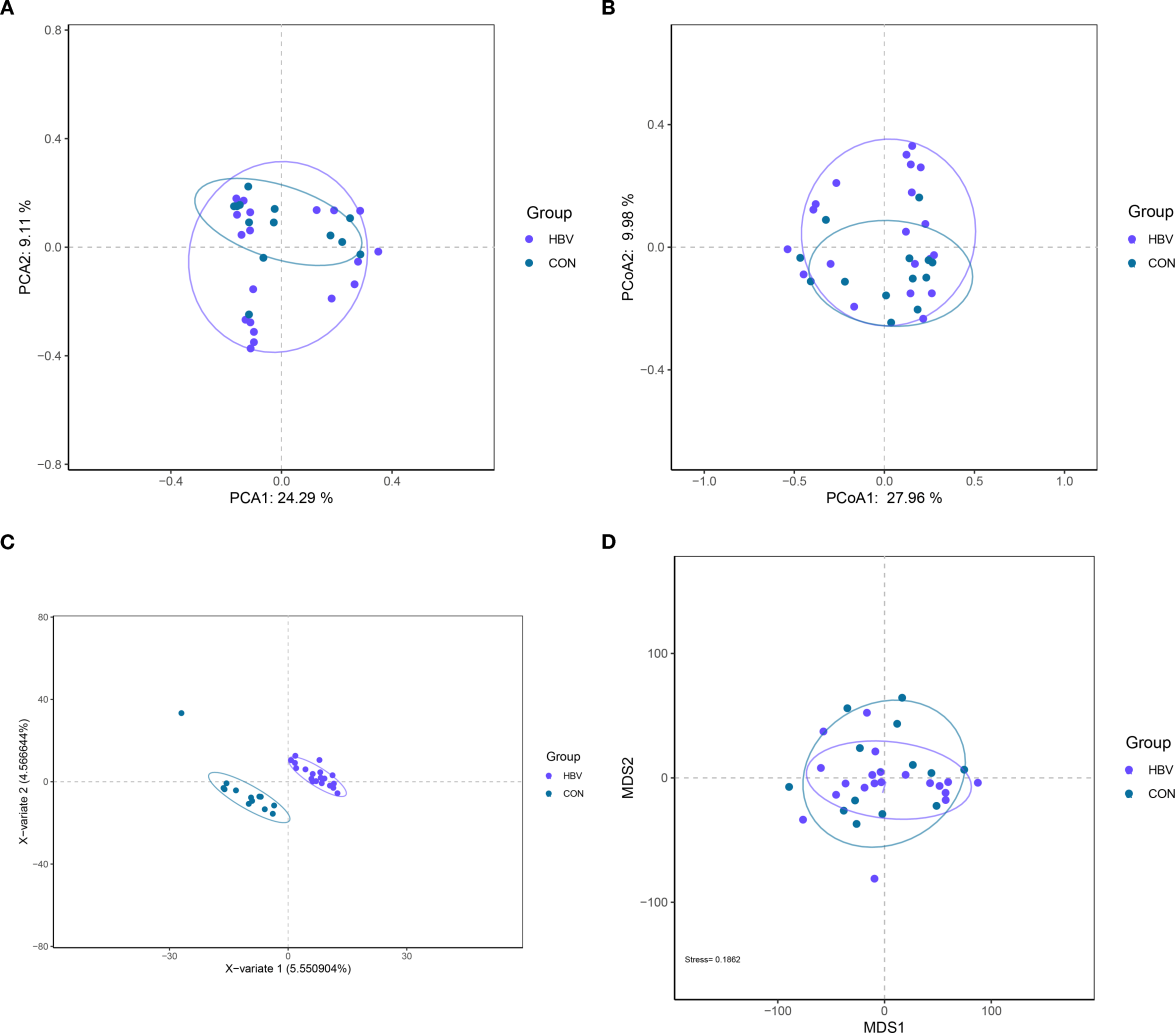
Beta diversity analysis of gut microbiota in the two groups. **(A)** PCA analysis; **(B)** PCoA analysis; **(C)** PLS-DA analysis; **(D)** NMDS analysis.

To assess differences in microbial communities between the two groups, beta diversity analysis was performed. While PCA and PCoA results showed partial overlap in gut microbial composition (**Figures 3A, B**), PLS-DA analysis effectively discriminated the groups into two distinct, non-overlapping clusters (**Figure 3C**). Additionally, the NMDS analysis showed a Stress value of 0.1862 (<0.2), reinforcing the reliability of the analysis and the structural divergence observed (**Figure 3D**). Collectively, these results indicate that pregnant HBV carriers possess a gut microbiota structure distinct from that of healthy controls.

### 3.5 Relative abundance of gut microbiota in the two groups

**Figure 4:**
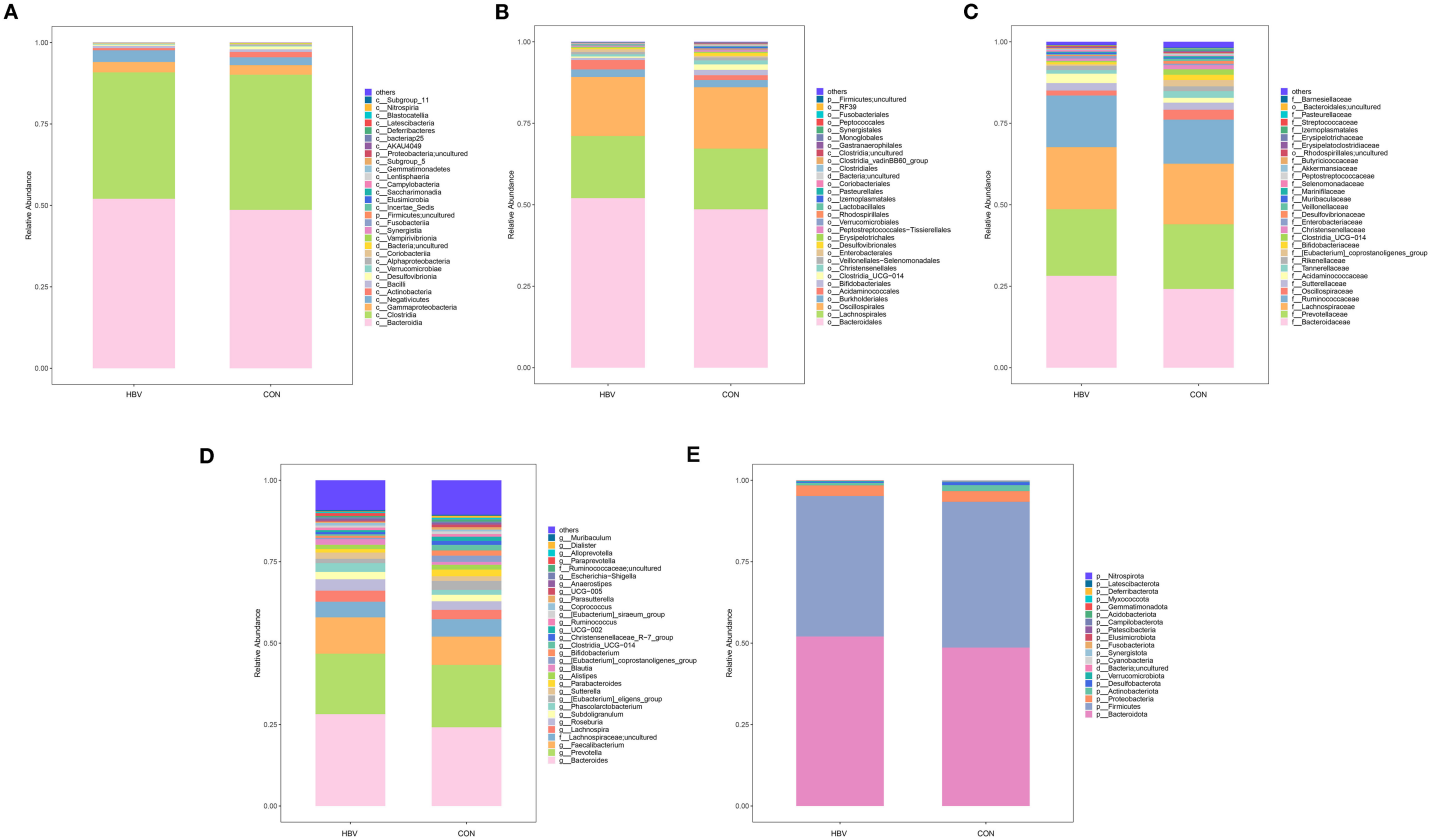
Relative abundance of gut microbiota at the class **(A)**, order **(B)**, family **(C)**, genus **(D)**, and phylum **(E)** levels in the two groups.

To investigate the changes in the gut microbial community in HBV-infected pregnant women, we analyzed the species composition at the Class, Order, Family, Genus, and Phylum levels. At the Class level, both the HBV and CON groups were dominated by Bacteroidia, Clostridia, and Gammaproteobacteria (**Figure 4A**). At the Order level, both groups were dominated by Bacteroidales, Lachnospirales, and Oscillospirales (**Figure 4B**). At the Family level, both groups were dominated by Bacteroidaceae, Prevotellaceae, and Lachnospiraceae (**Figure 4C**). At the Genus level, both groups were dominated by *Bacteroides*, *Prevotella*, and *Faecalibacterium* (**Figure 4D**). At the Phylum level, both groups were dominated by Bacteroidota, Firmicutes, and Proteobacteria (**Figure 4E**).

### 3.6 Analysis of species differences in gut microbiota between the two groups

**Figure 5:**
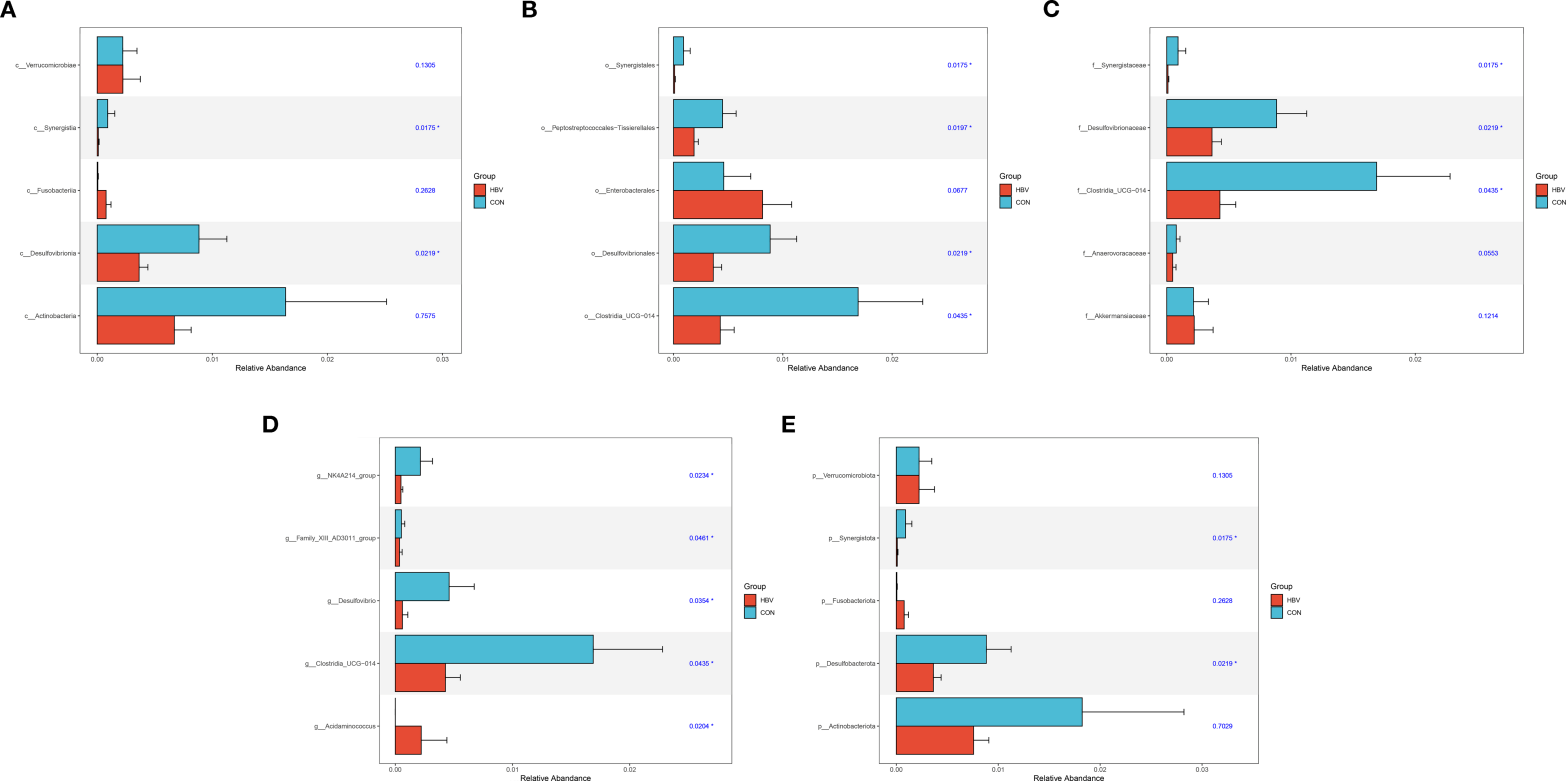
Analysis of species differences in gut microbiota at the class **(A)**, order **(B)**, family **(C)**, genus **(D)**, and phylum **(E)** levels between the two groups.

The Wilcoxon test was used for comparative analysis of gut microbiota between the CON and HBV groups. At the Class level, compared to the CON group, the HBV group showed significantly lower relative abundances of Synergistia and Desulfovibrionia (*p* < 0.05, **Figure 5A**). At the Order level, compared to the CON group, the HBV group showed significantly lower relative abundances of Synergistales, Peptostreptococcales-Tissierellales, Desulfovibrionales, and Clostridia_UCG-014 (an order-level uncultured clade within the class Clostridia) (*p* < 0.05, **Figure 5B**). At the Family level, compared to the CON group, the HBV group showed significantly lower relative abundances of Synergistaceae, Desulfovibrionaceae, and Clostridia_UCG-014 (a family-level uncultured clade within the class Clostridia) (*P* < 0.05, **Figure 5C**). At the Genus level, compared to the CON group, the HBV group showed significantly lower relative abundances of *NK4A214_group* (a genus-level uncultured clade within the family Oscillospiraceae), *Family_XIII_AD3011_group* (a genus-level uncultured clade within the family Family_XIII), *Desulfovibrio*, and *Clostridia_UCG-014* (a genus-level uncultured clade within the class Clostridia) (*p* < 0.05), and a significantly higher relative abundance of *Acidaminococcus* (*p* < 0.05, **Figure 5D**). At the Phylum level, compared to the CON group, the HBV group showed significantly lower relative abundances of Synergistota and Desulfobacterota (*p* < 0.05, **Figure 5E**).

### 3.7 Functional analysis of gut microbiota in the two groups

**Figure 6:**
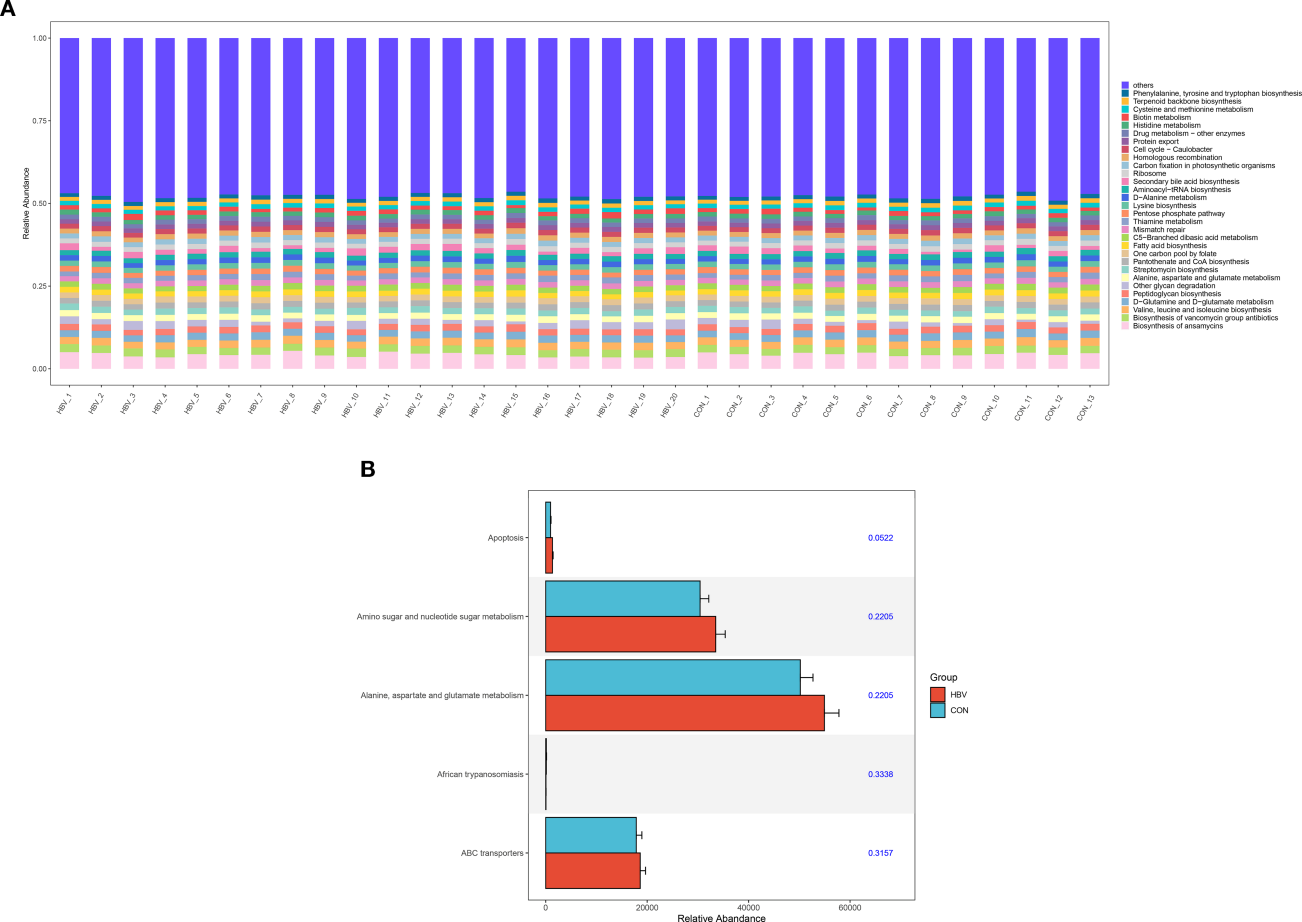
Functional analysis of gut microbiota in the two groups. **(A)** Bar chart of functional abundance. **(B)** Bar chart of significantly different functional genes.

The PICRUSt tool was used to predict the functional potential of the gut microbiota in the two groups. The functional composition of the gut microbiota in both groups is shown in **Figure 6A**. The most abundant functions and signaling pathways included Biosynthesis of ansamycins, Biosynthesis of vancomycin group antibiotics, Valine, leucine and isoleucine biosynthesis, D-Glutamine and D-glutamate metabolism, and Peptidoglycan biosynthesis. Furthermore, between-group comparison of significantly different functional genes between the two groups showed no statistically significant differences in the composition of bacterial functional genes in the fecal microbiota of the HBV group compared to the CON group (*p*>0.05, **Figure 6B**).

### 3.8 Correlation analysis between gut microbiota genera and liver biochemical indicators

**Figure 7:**
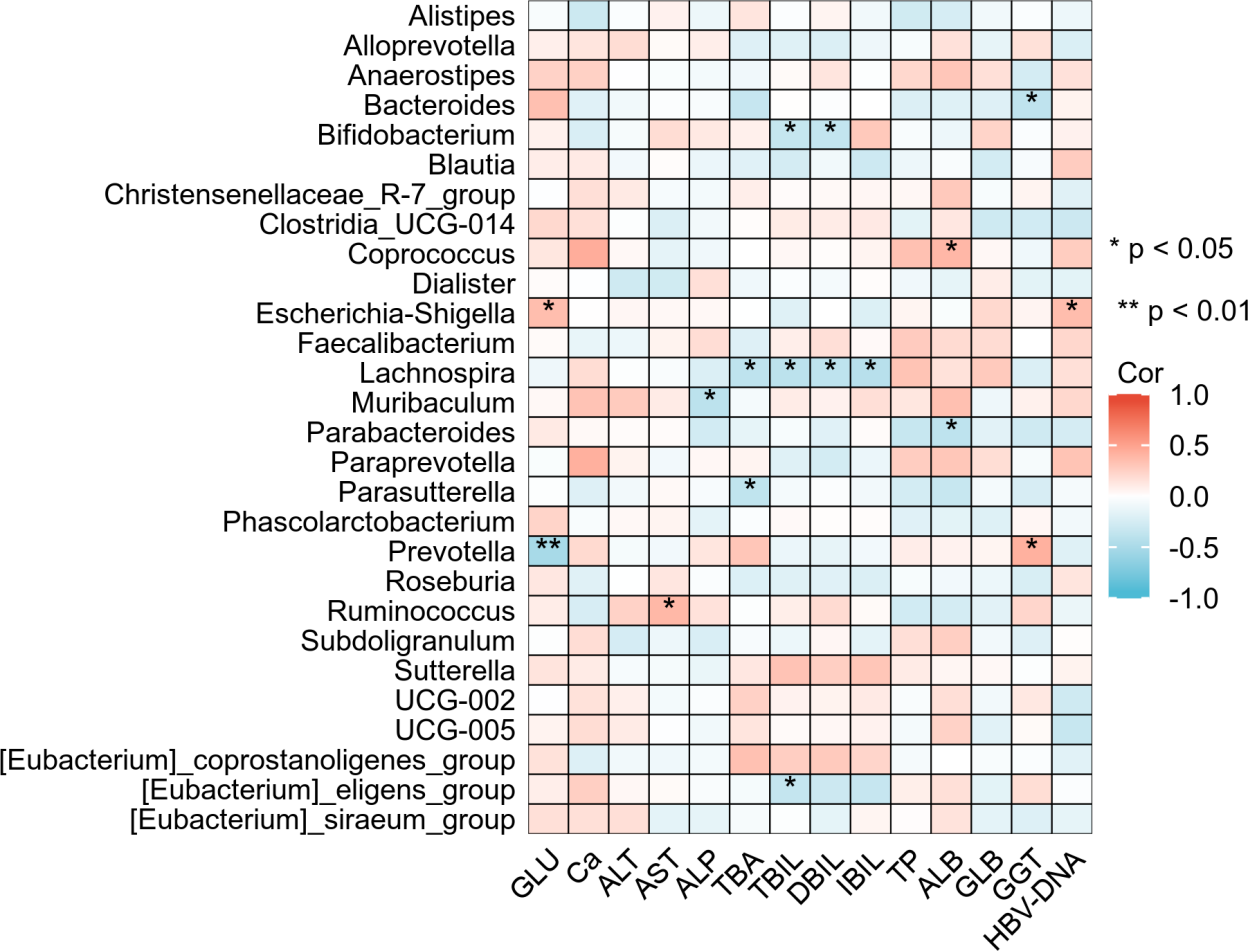
Correlation analysis between gut microbiota at the genus level and liver biochemical indicators. *p < 0.05, **p < 0.01.

Spearman correlation analysis was used to explore the associations between gut microbiota genera and liver biochemical indicators. The results are shown in **Figure 7**. *Bacteroides* was significantly negatively correlated with GGT (r=-0.384, *p* < 0.05). *Bifidobacterium* was significantly negatively correlated with TBIL (r=-0.351, *p* < 0.05) and DBIL (r=-0.362, *p* < 0.05). *Coprococcus* was significantly positively correlated with ALB (r=0.391, *p* < 0.05). *Escherichia-Shigella* was significantly positively correlated with GLU (r=0.355, *p* < 0.05) and HBV-DNA (r=0.360, *p* < 0.05). *Lachnospira* was significantly negatively correlated with TBA (r=-0.389, *p* < 0.05), TBIL (r=-0.402, *p* < 0.05), DBIL (r=-0.387, *p* < 0.05), and IBIL (r=-0.424, *p* < 0.05). *Muribaculum* was significantly negatively correlated with ALP (r=-0.403, *p* < 0.05). *Parabacteroides* was significantly negatively correlated with ALB (r=-0.387, *p* < 0.05). *Parasutterella* was significantly negatively correlated with TBA (r=-0.376, *p* < 0.05). *Prevotella* was significantly negatively correlated with GLU (r=-0.515, *p* < 0.01) and significantly positively correlated with GGT (r=0.426, *p* < 0.05). *Ruminococcus* was significantly positively correlated with AST (r=0.390, *p* < 0.05). *[Eubacterium]_eligens_group* was significantly negatively correlated with TBIL (r=-0.355, *p* < 0.05).

## 4 Discussion

HBV infection is a global epidemic and represents a major etiological factor in the development of liver fibrosis, cirrhosis, and hepatocellular carcinoma. It is reported that the HBV carrier state during pregnancy not only increases the burden on the liver, potentially leading to spontaneous preterm birth and recurrent abortion, but also increases the risk of neonatal infection through vertical transmission (Kushner et al., 2018). The gut microbiota, known as the “second genome,” participates in immune regulation, inflammatory responses as well as metabolic processes, and is closely associated with the progression of liver diseases (Fei et al., 2025). Therefore, in-depth analysis of the specific characteristics of the gut microbiota in pregnant HBV carriers and exploration of its correlation with liver injury are of great significance.

In the present study, we compared the differences in gut microbiota between pregnant HBV carriers and healthy pregnant women, and further investigated the correlation between liver biochemical parameters and the abundance of major gut bacterial genera. Although no significant between-group differences were observed in general characteristics, liver function-related indicators, or gut microbiota alpha diversity, beta diversity analyzes clearly indicated distinct differences in gut microbiota structure between the CON and HBV groups. Significant differences in relative abundance were observed at multiple taxonomic levels. Furthermore, significant correlations were observed between specific bacterial genera and liver function indicators.

### 4.1 Structural and diversity characteristics of gut microbiota in pregnant HBV carriers

Microbiota diversity serves as a key indicator reflecting the balance of the gut microecology. Alpha diversity reflects the richness and evenness of the microbiota within a sample, while beta diversity captures the degree of structural difference in microbiota between different groups (Cassol et al., 2025). In this study, there were no statistically significant differences in alpha diversity indices (ACE, Chao1, Shannon, etc.) between the HBV and CON groups. This differs from studies on non-pregnant HBV infected individuals, where alpha diversity is often reported to be reduced (Yang et al., 2023). This discrepancy might be related to the special physiological state of pregnancy. Pregnant women themselves undergo gut microbiota remodeling due to hormonal changes, immune suppression, and other factors, often accompanied by reduced diversity (Giugliano et al., 2025). The HBV carrier state might not further exacerbate the change in alpha diversity on this basis, or the effects of the two factors might offset each other, ultimately showing no significant changes in alpha diversity. Additionally, the relatively small sample size in this study might have limited the power to detect significant differences in alpha diversity.

The results of beta diversity analysis provide key evidence for the unique gut microbiota structure in pregnant HBV carriers. PCA and PCoA analyzes showed partial overlap in microbiota composition between the two groups, while PLS-DA analysis completely separated the samples into two distinct clusters. The NMDS analysis Stress value < 0.2 also confirmed the reliability of the results. This indicates that although the overall richness and evenness of the microbiota are similar between the groups, there are significant differences in the specific composition and proportions of species, suggesting that HBV infection is associated with differences in gut microbiota composition within the unique physiological context of pregnancy. Similar findings have been reported in studies on patients with chronic hepatitis B, where beta diversity was more sensitive than alpha diversity in reflecting HBV-associated gut microbiota dysbiosis (Lin et al., 2023). Our results further confirm this characteristic in the pregnant population.

### 4.2 Compositional and functional changes in gut microbiota of pregnant HBV carriers

Regarding the core dominant microbiota, this study demonstrated that the main dominant taxa at the Phylum, Class, Order, Family, and Genus levels were largely consistent between the two groups. Bacteroidota, Firmicutes, and Proteobacteria were the dominant phyla, while Bacteroidia and Clostridia were the dominant classes. This aligns with previous findings on the core composition of gut microbiota in pregnant women (Yao et al., 2025). We propose that HBV carriage during pregnancy does not disrupt the core ecological structure of the gut microbiota but selectively affects the abundance of non-dominant bacterial groups. This could be a result of adaptive regulation by the body to maintain normal pregnancy physiology.

In the differential species analyzes, the relative abundances of several taxonomic units were significantly lower in the HBV group, including Synergistota and Desulfobacterota at the phylum level, Synergistia and Desulfovibrionia at the class level, and Synergistales and Desulfovibrionales at the order level. Conversely, the abundance of the genus *Acidaminococcus* was significantly increased. Bacteria within Synergistota and Desulfobacterota are typically anaerobic fermenters involved in the degradation of proteins and amino acids and the synthesis of short-chain fatty acids (SCFAs) in the gut. Their reduced abundance might lead to disrupted nitrogen metabolism and decreased SCFA production (Duncan et al., 2023). SCFAs, as important metabolites of gut microbiota, can inhibit the hepatic NF-κB inflammatory pathway by activating GPR41/43 and promote hepatic fatty acid oxidation. A decrease in SCFA levels might weaken their anti-inflammatory and metabolic protective effects on the liver, posing potential risks for subsequent liver dysfunction (Li X. et al., 2024). *Desulfovibrio*, a representative genus of Desulfobacterota, is involved in regulating intestinal redox balance through the production of hydrogen sulfide and is closely related to intestinal barrier function (Lu and Wen, 2025). Some studies have shown that increased abundance of *Desulfovibrio* in patients with chronic cirrhosis is closely associated with increased intestinal permeability and endotoxemia (Lu et al., 2023). Contrary to expectations, our study found a significant decrease in the relative abundance of *Desulfovibrio* in pregnant HBV carriers. Since the proliferation of *Desulfovibrio* depends on a mildly inflammatory and sulfate-rich environment, the generally anti-inflammatory environment during pregnancy might selectively suppress this genus (Singh et al., 2022). However, whether the reduction of *Desulfovibrio* affects intestinal barrier function in pregnant HBV carriers needs further verification with indicators such as intestinal permeability tests. Furthermore, the significant increase in *Acidaminococcus* abundance in the HBV group is notable, as this genus is involved in branched-chain amino acid (BCAA) metabolism. Disrupted BCAA metabolism is closely related to abnormal liver synthetic function, suggesting that changes in its abundance might be an important marker of adaptive metabolic adjustments in the livers of pregnant HBV carriers (Trillos-Almanza et al., 2024).

PICRUSt functional prediction results showed that the most abundant pathways in the gut microbiota of both groups were concentrated in vancomycin group antibiotic biosynthesis, valine, leucine, and isoleucine biosynthesis, peptidoglycan biosynthesis, etc. These findings suggest that the gut microbiota during pregnancy may play an important role in maintaining host amino acid metabolism and cell wall homeostasis, consistent with the observations reported by Giugliano et al (Giugliano et al., 2025). Additionally, the composition of core predicted metabolic pathways did not differ significantly between the two groups, suggesting that despite the observed differences in gut microbiota composition between the HBV and CON groups, the core metabolic functions were similar in this cohort. This characteristic corresponds to the absence of significant difference in liver function indicators between the groups, suggesting that the homeostasis of gut microbiota function might be an important guarantee for maintaining normal liver function in pregnant HBV carriers. In the study by Saleh et al., the comparison of ALT, AST and other liver function markers between HBV carriers and healthy controls showed no statistically significant difference, consistent with our results (Saleh, 2015).

### 4.3 Correlation analyzes between gut microbiota and liver function/HBV replication

Spearman correlation analyzes results showed that *Bacteroides* was significantly negatively correlated with GGT. GGT is a specific indicator of hepatobiliary duct injury, and its level is closely related to disordered bile acid metabolism (Gerussi, 2022). *Bacteroides* can hydrolyze conjugated bile acids into free bile acids by secreting bile salt hydrolases, promoting the enterohepatic circulation of bile acids (Zhang et al., 2022). A decrease in *Bacteroides* abundance was correlated with changes in bile acid metabolism-related indicators, suggesting a potential association with hepatobiliary function. *Bifidobacterium*, *Lachnospira*, and *[Eubacterium]_eligens_group* were all negatively correlated with bilirubin indicators such as TBIL and DBIL, suggesting these genera might be involved in the regulation of bilirubin homeostasis during pregnancy by promoting bilirubin conjugation and excretion, thus influencing liver function (Li et al., 2023; Li J. et al., 2024). Hu et al (Hu et al., 2022). likewise reported a positive correlation between *Coprococcus* and ALB. ALB is a core indicator reflecting liver synthetic function, and its association with *Coprococcus* might stem from the support that *Coprococcus*-involved amino acid metabolism provides for liver synthetic function (Notting et al., 2023). Conversely, the negative correlation between *Parabacteroides* and ALB suggests that its abnormal abundance might impair liver synthetic capacity (Dong et al., 2020). Furthermore, we found that *Escherichia-Shigella* was significantly positively correlated with HBV DNA load. An increased abundance of *Escherichia-Shigella* is often accompanied by impaired intestinal barrier and endotoxin entry into the bloodstream. It has been reported that endotoxin may be associated with HBV replication by activating the TLR4/NF-κB pathway (Kang et al., 2023; Wang Y. et al., 2025). The dual association of *Prevotella* (a negative correlation with GLU and a positive correlation with GGT) suggests its potential involvement in both glucose metabolism and bile acid metabolism, implying a complex role in the metabolic homeostasis of pregnant HBV carriers (Precup and Vodnar, 2019; Pinto et al., 2023). The positive correlation between *Ruminococcus* and AST suggests a potential association with hepatocyte-related biochemical changes by releasing pro-inflammatory metabolites (Jiao et al., 2022). The negative correlation between *Parasutterella* and TBA hints at its potential role in alleviating bile acid toxicity (Guo et al., 2018). Taken together, these results collectively construct a complex network of associations between gut bacterial genera and liver function indicators, highlighting potential targets and providing a theoretical basis for intervening in liver diseases through the regulation of gut microbiota.

### 4.4 Conclusion and prospects

In summary, significant differences were observed in the gut microbiota structure between pregnant HBV carriers and healthy pregnant women, and specific bacterial genera are closely related to liver biochemical indicators and HBV DNA load. These findings suggest potential associations between gut microbiota and HBV infection during pregnancy, and highlight the microbiome as a potentially valuable target for clinical risk assessment in HBV infection during pregnancy. However, this study has limitations. Firstly, this work is explicitly described as a pilot study and an initial phase of an ongoing research project. The sample size is modest, which may limit generalizability. It is important to note that the current samples were collected over a considerable period of time due to difficulties in sample acquisition. Despite this limitation, we have obtained some meaningful and statistically significant findings. Secondly, key clinical variables-including HBeAg status, history of antiviral therapy, HBV genotype, duration of infection, history of acute ALT flares, and liver fibrosis assessment-were not systematically recorded. As these patients were true asymptomatic carriers with no indications for antiviral treatment, such detailed infection status data were not systematically collected in this retrospective pilot study, which may affect the interpretation of the associations between gut microbiota and HBV infection status. Thirdly, several potential confounding factors (e.g., dietary intake, probiotic or fermented food consumption, constipation status, and socioeconomic factors) were not controlled for, and the limited sample size precluded multivariate adjustment for confounders such as age, gestational age, and pre-pregnancy BMI. Fourthly, the absence of a non-pregnant HBV-infected control group limited our ability to fully dissect the interaction between pregnancy-related microbiota remodeling and HBV infection. Future studies should expand the sample size, incorporate comprehensive clinical phenotyping, and include appropriate control groups to validate these preliminary findings.

## Data Availability

The original contributions presented in the study are included in the article/supplementary material. Further inquiries can be directed to the corresponding author.
